# The Familial Burden: Recognizing Obsessive-Compulsive Disorder Through Proxy Family Members

**DOI:** 10.7759/cureus.78801

**Published:** 2025-02-10

**Authors:** Varchasvi Mudgal, Priyash Jain, Koustubh R Bagul, Sanjay Prasad

**Affiliations:** 1 Psychiatry, Mahatma Gandhi Memorial Medical College, Indore, IND; 2 Psychiatry, Bundelkhand Medical College, Sagar, IND

**Keywords:** consultation liaison psychiatry, family accommodation, obsessive-compulsive disorders, ocd and related disorders, psychiatric disorders/mental disorders

## Abstract

The study highlights six cases in which obsessive-compulsive disorder (OCD) in a primary patient was recognized through distress or behavioral changes in a family member. Common themes included compulsive behaviors that led to physical and emotional strain on relatives, leading them to seek medical attention. This underscores the indirect impact of OCD on family members, emphasizing the bio-psycho-social interplay in symptom manifestation and caregiving dynamics. Familial accommodation often perpetuates compulsions, as seen in caregivers assuming proxy roles. Genetic predisposition, cognitive distortions, and cultural influences shape OCD's expression and management. Interventions such as psychoeducation and family-based cognitive-behavioral therapy (CBT) can reduce family accommodation, fostering better outcomes. The cases underscore the importance of systemic approaches, early diagnosis, and addressing caregiver burden to mitigate OCD's ripple effects, emphasizing the need for holistic and family-inclusive care.

## Introduction

Obsessive-compulsive disorder (OCD) is a chronic, debilitating mental health condition characterized by intrusive, unwanted thoughts (obsessions) and repetitive behaviors or mental acts (compulsions) intended to reduce the distress caused by these thoughts. Affecting approximately 2.5% of the population, OCD significantly impairs personal, social, and occupational functioning, often resulting in considerable morbidity [1]. However, its impact often extends beyond the individual, profoundly affecting families, caregivers, and close associates, who may experience its effects even more acutely than the person with OCD [2]. In particular, accommodating behaviors by family members can inadvertently exacerbate or enable obsessive symptoms [3].

Interestingly, the disorder is sometimes identified not through the symptoms of the affected individual but through the distress experienced by their close contacts. This case series highlights six instances where OCD was indirectly recognized via its impact on family members or close associates, referred to here as proxies. These proxies presented with diverse physical, psychological, or behavioral challenges, which were eventually traced back to OCD symptoms in a family member. For example, children may become direct participants in rituals, spouses may unintentionally enable compulsions, or parents may mirror obsessive behaviors, demonstrating the pervasive influence of OCD. In rare cases, prolonged exposure to OCD-related behaviors has even led proxies to develop similar symptoms themselves [4].

The diagnosis of OCD in these cases underscores the importance of detailed family and social histories, particularly in cultural contexts where certain behaviors may be normalized, delaying recognition and treatment. By highlighting this phenomenon, the case series emphasizes the need for heightened clinical awareness of how OCD symptoms can manifest indirectly through family dynamics. Early recognition and intervention can alleviate distress for individuals with OCD and their proxies, ensuring better outcomes for all involved. The study aimed to explore the recognition of OCD through family members who presented with distress linked to an undiagnosed OCD case in a close relative.

## Materials and methods

This case series was conducted at the Department of Psychiatry, MGM Medical College and associated hospitals. Cases were identified through clinical evaluations conducted at the Psychiatry Outpatient Department (OPD) over an interval of one year. Patients were selected based on the criterion that OCD in the primary patient was recognized through distress or symptoms in a family member (proxy). The diagnosis of OCD was made by a trained psychiatrist based on the International Classification of Diseases Version 10 (ICD-10). Confidentiality was maintained by anonymizing patient identifiers.

## Results

Case one

An eight-year-old boy presented to the Department of Pediatrics with recurrent episodes of gastric discomfort, which led to a diagnosis of gastric ulcers. Concern arose during discussions about his eating habits when his father revealed the possibility of phenyl disinfectant contamination in the boy's water bottle and utensils, which had been excessively cleaned by his mother. Parents were subsequently requested to visit the Psychiatry OPD for further evaluation.

The boy's mother, a 39-year-old homemaker, was found to have severe contamination obsessions and cleaning compulsions, characteristic of OCD. Her intense fear of germs harming her family drove her to compulsively mix phenyl with water to clean surfaces, including her son’s school supplies. Despite no prior psychiatric history or significant stressors, her cleaning behaviors were excessive, irrational, and disruptive to her family’s well-being. On mental status examination, she was found to be having obsessions regarding contamination of household utensils with germs along with compulsive cleaning with phenyl.

She was diagnosed with OCD and initiated on fluoxetine 20 mg, which was optimized to 40 mg alongside psychoeducation. Fluoxetine was further optimized up to 60 mg in the next follow at one-month intervals and within the next three months, her compulsive behaviors diminished as her Yale-Brown Obsessive Compulsive Scale (Y-BOCS) score dropped from 27 to 12, and the boy’s gastric symptoms resolved.

Case two

A five-year-old girl presented to the dermatology OPD with severe dryness and peeling of the skin on her hands. Initially suspected to have eczema or allergic dermatitis, further history-taking revealed frequent handwashing as the cause. Her mother insisted on washing the child’s hands over 20 times daily, driven by an intense fear of contamination. The mother was subsequently advised to get a Psychiatric opinion, after which she presented to the psychiatry OPD.

The mother, a 35-year-old homemaker from a semi-urban area, exhibited contamination obsessions and compulsive behaviors rooted in irrational fears that her daughter could contract harmful germs. On detailed evaluation, it was revealed that the complaints started five months back when her daughter caught flu. After this, she started cleaning her hands excessively sometimes three to four times in a single instance. This compulsive behavior not only caused physical harm to the child but also created emotional stress within the family. The mother had no prior personal or family history of psychiatric disorders, and her background indicated a stable life with no significant stressors.

Diagnosed with OCD, the mother began treatment with fluoxetine up to 40 mg and psychoeducation to address her distorted beliefs about contamination. Cognitive-behavioral therapy (CBT) with exposure and response prevention (ERP) was also initiated. Over three months, her compulsions significantly reduced, and her daughter’s dermatological condition resolved.

Case three

A 38-year-old woman sought help due to increasing distress caused by her husband’s unusual behaviors, which had severely disrupted their daily lives and strained their marriage. Her husband, a 41-year-old white-collar professional, exhibited obsessive fears of losing personal belongings during visits to others’ homes. These obsessions led to compulsive behaviors, such as repeatedly returning to visited homes, often late at night, to ensure nothing was left behind. He frequently insisted that his wife accompany him, intensifying her frustration and concern.

Although the man acknowledged the irrationality of his actions, he felt powerless to stop them. He had no prior psychiatric history, and his family history was unremarkable. His personal history indicated a stable upbringing and successful employment. However, over the past year, his symptoms had progressively worsened, significantly affecting his work performance and marital relationship. His wife would often get frustrated trying to assuage his concerns about having left personal belongings at the others’ home. Over the course of the next few months, the couple went through a lot of many altercations, which affected their relationship and subsequently prodded the wife to seek psychiatric help for herself. The evaluation of the wife’s source of distress led to the discovery of her husband’s psychiatric disorder who was subsequently reassured to seek consultation.

The husband was then diagnosed with OCD, he was started on Escitalopram 10 mg, which was optimized up to 40 mg in subsequent follow-ups. CBT with a focus on ERP was also initiated and over six months, his compulsive checking behaviors reduced significantly, and he gained better insight into his condition. His wife also experienced relief as their relationship improved.

Case four

A 50-year-old mother sought psychiatric help due to the profound physical and emotional strain caused by her son’s compulsive behaviors. Her 20-year-old son, a college student, exhibited obsessions centered on blasphemy and an intense fear that religious transgressions could harm his family. These obsessions drove him to compel his mother to visit a temple five times daily to offer offerings to the priest, believing any lapse would lead to severe consequences such as illness or death in the family.

Out of concern for her family’s well-being, the mother complied, despite significant disruption to her daily life, physical exhaustion, and emotional distress. A psychiatric evaluation identified her son as the source of the issue, and he was then advised to visit with his mother during the follow-up. Later, upon detailed evaluation, he elaborated on having repeated images of having committed a sacrilegious act, which he believed would result in God punishing his family. He also reported feelings of guilt and anxiety, and as a result, kept making his mother perform all the rituals to placate God.

Diagnosed with OCD, he began treatment with fluoxetine, optimized up to 60 mg, and ERP therapy. Over four months, his symptoms improved significantly, reducing his compulsive behaviors and reliance on his mother. The mother, in turn, experienced relief.

Case five

A nine-year-old girl from a rural school was referred for psychiatric evaluation after her teachers observed unusual behaviors for about a month. She repeatedly retrieved her assignments from the class teacher to draw symmetrical lines, expressing intense fear that any asymmetry could lead to catastrophic consequences. Upon further inquiry, it emerged that these behaviors were influenced by her 14-year-old brother, who exhibited obsessions with symmetry and compulsions to align objects perfectly for about eight to nine months. He frequently warned his younger sister about dire outcomes if symmetry was not maintained, inadvertently transmitting his fears to her.

The boy had never been diagnosed with a psychiatric condition, and he expressed to have been experiencing significant anxiety when things were misaligned. His developmental and family history were unremarkable. His sister had internalized his compulsions, leading to their independent manifestation, particularly in academic settings.

The boy was diagnosed with OCD and initiated on fluoxetine 20 mg complemented by CBT with ERP. His symptoms improved significantly. The younger sister underwent psychoeducation about her brother’s illness and brief psychotherapeutic interventions to address her emerging OCD. Psychoeducation helped the family minimize unintentional reinforcement of OCD-like behaviors and fostered a supportive environment.

Case six

A 10-year-old girl was referred to the Child Guidance Clinic through the department of Pediatrics by her school counselor due to declining academic performance and refusal to complete assignments. Teachers noted her insistence on counting words repetitively in essays and erasing sentences unless they contained "safe numbers" (e.g., multiples of three). During the evaluation, she revealed that her 16-year-old sister compelled her to count household items (e.g., stairs, cutlery) daily, warning that "something terrible" would happen if counts were inaccurate. 

The older sister, initially resistant to evaluation, admitted to severe counting obsessions (e.g., needing to count breaths to prevent family harm) and compulsions lasting four to six hours daily. She scored 32 on the Y-BOCS, meeting the criteria for severe OCD. Treatment included sertraline (titrated to 150 mg) and ERP targeting counting rituals. The younger sister received psychoeducation to disentangle her participation in rituals. At the four-month follow-up, the older sister’s Y-BOCS score dropped to 18, and the younger sister resumed normal academic engagement.

A summary of OCD cases identified through proxy family members is presented in Table 1.

**Table 1:** Summary of OCD cases identified through proxies OCD: obsessive-compulsive disorder; CBT: cognitive-behavioral therapy; ERP: exposure and response prevention

Case	The primary reason for consultation	Contact department	Source of the problem	Treatment
1	Recurrent gastric ulcers in 8-year-old boy	Pediatrics	39-year-old Mother's contamination obsessions (cleaning with phenyl)	Fluoxetine (20→60 mg)+psychoeducation
2	Dry, peeling skin on hands in 5-year-old girl	Dermatology	35-year-old Mother's compulsive handwashing of child	Fluoxetine (20→40 mg)+CBT/ERP
3	Marital discord and distress in 38-year-old wife	Psychiatry	41-year-old Husband's obsessive fears about leaving items behind and checking for items left	Escitalopram (10→40 mg)+CBT/ERP
4	Distress in 50-year-old mother	Psychiatry	20-year-old son’s obsessive fears of blasphemy; compelled mother to perform rituals	Fluoxetine (20→60 mg)+ERP
5	Unusual behavior by a 9-year-old girl	School counsellor	14-year-old brother’s obsessions with symmetry; sister mimicking brother's compulsions	Fluoxetine (20 mg)+CBT/ERP
6	Academic denial in 10-year-old girl	Pediatrics	Counting OCD in 16-year-old sister	Sertraline (50→150 mg)+CBT/ERP

## Discussion

The six cases presented in this series illustrate how OCD can indirectly manifest through proxies, demonstrating its potential to disrupt relationships, burden caregivers, and influence behavior in unintended ways. This discussion explores the bio-psycho-social underpinnings of these cases and their clinical implications.

Biological factors

OCD is associated with abnormalities in the cortico-striato-thalamo-cortical loop, with genetic predisposition playing a significant role. Studies indicate that family members of individuals with OCD are at a higher risk of developing the disorder. This is evident in Case Five, where the sister’s compulsive behaviors mimicked her brother’s, eventually resulting in her own diagnosis of OCD. The interplay of genetic susceptibility and learned behaviors is underscored here [5,6].

Psychological factors

OCD is driven by heightened sensitivity to intrusive thoughts, exaggerated risk perceptions, and an overwhelming need for control. These psychological traits were evident in all cases. In Case Three, the husband’s obsessive fear of losing belongings led to repeated home checks, reflecting a distorted sense of responsibility. Similarly, in Case Four, a 20-year-old exhibited cognitive distortions related to blasphemy, compelling his mother to perform religious rituals under duress.

The emotional dynamics of guilt, fear, and coercion often played a role in proxy involvement. For instance, in Case Two, a mother compulsively washed her daughter’s hands to protect her from perceived contamination, inadvertently causing dermatological issues in the child. These cases highlight the importance of psychoeducation in helping families understand and address the irrationality of OCD-driven fears [7].

Social factors

The social impact of OCD was evident in strained familial relationships and patterns of family accommodation (FA), where relatives adjusted their behaviors to alleviate the patient’s distress. This often perpetuated compulsions, as seen in Case Four, where a mother’s compliance with her son’s demands reinforced his rituals [8].

Cultural factors and social stigma also shaped OCD presentations and management. For instance, the compulsive rituals in Case Four might have been normalized in a religiously inclined community, delaying diagnosis. Similarly, in Cases One and Two, societal expectations of maternal responsibility obscured the pathological nature of caregiving behaviors, prolonging distress for both the mothers and their children.

Similarly, in Case Six, it is similarly demonstrated how sibling dynamics can transmit OCD symptoms. The proxy’s academic refusal stemmed from internalizing her sister’s counting compulsions, illustrating how OCD has both biological and environmental roots.

Family accommodation and treatment implications

FA in OCD, while often driven by empathy and dependency, reinforces compulsions and hinders treatment outcomes, especially in CBT. Non-compliance by the family members may result in conflicts and subsequently distress among the family members. Tools like the Family Accommodation Scale (FAS) can identify the extent of FA, enabling targeted interventions to improve outcomes for patients and their caregivers. Psychoeducation and family-based CBT reduce FA by equipping families with constructive support strategies.

These cases emphasize the importance of systemic approaches to OCD diagnosis and treatment. Early intervention mitigated the secondary effects of OCD on family members, as seen in Cases One and Two, where timely treatment resolved symptoms in both children and their proxies. However, Case Five and Case Six underscore the challenges of managing familial OCD, highlighting the potential for symptom transmission through observational learning and environmental factors [9,10]. Early school-based interventions, combined with family psychoeducation, disrupted this cycle, aligning with findings on the efficacy of systemic approaches.

Finally, these cases underscore the caregiver burden of OCD and the necessity of integrating caregiver mental health into therapeutic processes. While the outcomes were positive, further research with larger sample sizes and long-term follow-ups could expand our understanding of OCD’s indirect manifestations. Also, the case series highlights the need to keep a holistic approach to managing patients in a comprehensive way.

Limitations

Due to its case series design, this study is limited by its small sample size and lack of a control group. The absence of long-term follow-up data restricts conclusions about the sustained impact of interventions. As cases were managed on an OPD basis many of them have missing data on instruments like Y-BOCS and FAS scale. Further studies with larger sample sizes are warranted to validate these findings.

## Conclusions

This case series highlights the importance of recognizing OCD through its impact on family members. Early identification and intervention can alleviate distress for both the patient and their relatives. A family-centered approach, incorporating psychoeducation and behavioral interventions, is crucial in improving outcomes and reducing caregiver burden. 
